# Revisiting the miR-200 Family: A Clan of Five Siblings with Essential Roles in Development and Disease

**DOI:** 10.3390/biom12060781

**Published:** 2022-06-03

**Authors:** Vignesh Sundararajan, Ulrike C. Burk, Karolina Bajdak-Rusinek

**Affiliations:** 1 Cancer Science Institute of Singapore, National University of Singapore, Center for Translational Medicine, Singapore 117599, Singapore; csivsun@nus.edu.sg; 2 Institute of Molecular Medicine and Cell Research, Faculty of Medicine, University of Freiburg, 79104 Freiburg, Germany; ulrike.burk@gmx.de; 3 Department of Medical Genetics, Faculty of Medical Sciences, Medical University of Silesia, 40-752 Katowice, Poland

**Keywords:** miRNA/microRNA, miR-200 family, cancer-associated miRNAs, epithelial-to-mesenchymal transition (EMT), development, neurodegenerative diseases

## Abstract

Over two decades of studies on small noncoding RNA molecules illustrate the significance of microRNAs (miRNAs/miRs) in controlling multiple physiological and pathological functions through post-transcriptional and spatiotemporal gene expression. Among the plethora of miRs that are essential during animal embryonic development, in this review, we elaborate the indispensable role of the miR-200 family (comprising miR-200a, -200b, 200c, -141, and -429) in governing the cellular functions associated with epithelial homeostasis, such as epithelial differentiation and neurogenesis. Additionally, in pathological contexts, miR-200 family members are primarily involved in tumor-suppressive roles, including the reversal of the cancer-associated epithelial–mesenchymal transition dedifferentiation process, and are dysregulated during organ fibrosis. Moreover, recent eminent studies have elucidated the crucial roles of miR-200s in the pathophysiology of multiple neurodegenerative diseases and tissue fibrosis. Lastly, we summarize the key studies that have recognized the potential use of miR-200 members as biomarkers for the diagnosis and prognosis of cancers, elaborating the application of these small biomolecules in aiding early cancer detection and intervention.

## 1. Introduction

In classical molecular biology, the flow of genetic information from DNA to messenger RNA (mRNA) and thereafter to protein is regarded as the “central dogma” of life. However, the fraction of protein-coding genes accounts for merely ~3% of the human genome, whereas almost 97% of the genome is non-protein coding, which remained puzzling for several years. Until the early 1970s, this bulk of genetic material was largely considered to be functionally inactive and even termed as “junk DNA” [1]. However, with the advent of systemic whole-genomic sequencing analyses and extensive genome-wide association studies, thousands of “silent” genetic elements such as cis/trans-gene-regulatory elements, introns, repetitive sequences, transposable elements, telomeres, and pseudogenes were identified and functionally characterized within the so-called “junk DNA”. Such non-protein-coding, gene regulatory elements were attributed as one of the paramount discoveries in genomics ever since the decoding of the DNA structure in 1953 [2,3,4]. Further exploration of these non-protein-coding segments of the genome showed that a significant portion of these genes are transcribed as non-coding RNA (ncRNA), which are capable of influencing multiple and highly specific biochemical and molecular processes such as chromatin accessibility, RNA splicing, and transcription and/or protein translation-rate determination. Besides the fundamental ncRNAs (transfer RNA (tRNA) and ribosomal RNA (rRNA)) that are integral to the central dogma, eukaryotic genomes encode for equally important classes of ncRNAs such as microRNAs (miRNA/miR), small interfering RNAs (siRNA), small nuclear RNAs (snRNA), Piwi-interacting RNAs (piRNA), and long non-coding RNAs (lncRNA).

**Figure 1:**
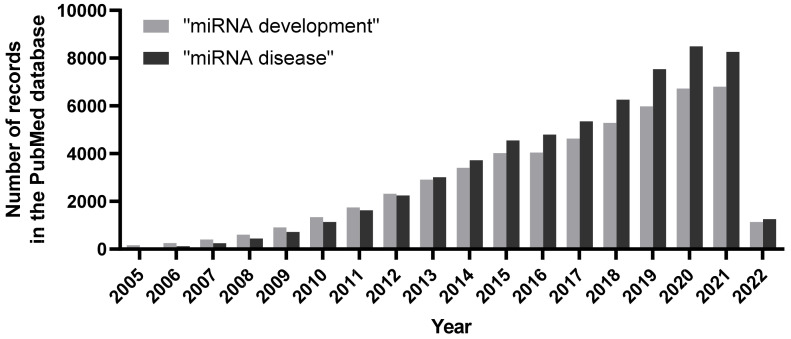
Number of publications per year associated with search terms “miRNA development” and “miRNA disease” in the PubMed database. An increasing trend of publications unearthing miRNAs and their roles during organismal development, as well as during pathological scenarios, clearly denote their vital biological implications.

MiRNAs/miRs are a prominent subclass of single-stranded ncRNAs, whose transcripts are about 19–22 nucleotides in length. MiRNAs bind to the 3′ untranslated region (3′UTR) of target mRNAs to elicit mRNA degradation and/or protein translational repression [5]. The most salient feature of miRNA–mRNA targeting is the presence of a 7 nt “seed” region (nucleotides 2–8) in every miRNA that exhibit sequence complementarity to its target mRNA, among which the complementarity of the seed sequence is absolutely crucial for target specificity [6]. According to the recent release, the miRBase database (http://mirbase.org/, accessed on 1 June 2022, exclusive public repository for miRNA sequences and annotation) contains over 38,000 precursors and 48,000 mature miRNA entries from 271 organisms [7]. RNA polymerase II transcribes miRs in the nucleus to form pri-miR transcripts. Next, the RNase III enzyme Drosha processes pre-miRNA transcripts into ~70-nucleotide pre-miR with imperfect stem-loop structures, which is exported into the cytoplasm. Subsequently, Dicer (another RNase III enzyme) processes the pre-miRNA and generates a transient ~22-nucleotide miRNA: miRNA* duplex molecule. The miRNA: miRNA* duplex is incorporated into the miRISC complex, which includes the Argonaute proteins and the RNA-binding protein TRBP. The mature miR strand is preferentially retained in the functional miRISC complex. The miRISC complex finally delivers the mature miR to its target mRNA and mediates the site-specific cleavage and degradation of the mRNA or inhibits its translation [8]. In addition to the canonical miRNA targeting mechanism, recent small RNA deep-sequencing data reveal the presence of differential and/or the distinct subcellular localization of miRNAs to execute unconventional functions in cellular homeostasis [9,10,11,12]. Based on the large volume of studies investigating miRs, it is fair to presume that miRs have equally indispensable roles during development and in a variety of human diseases (Figure 1).

The human genome encodes for more than 2500 mature miRNAs that exert their functions in during several physiological processes such as embryogenesis, muscle differentiation, and stem cell division, as well as in numerous pathological conditions such as cardiovascular disease, autoimmune disease, and cancer [7,13]. As far as the role of miRNAs in cancer is concerned, these molecules can variably function either as tumor suppressors or as oncogenes. As tumor suppressors, miRNAs regulate the expression of several oncogenic factors. For example, miR-15a and miR-16-1 post-transcriptionally downregulate B cell lymphoma 2 (BCL2), an anti-apoptotic gene that is often overexpressed in different types of leukemia and lymphomas [14]. Significant reductions of miR-143 and miR-145 are observed in colorectal cancers, denoting that the expression of certain miRNAs is abrogated during carcinogenesis [15]. As oncogenes, the aberrant activation of certain miRNAs could directly or indirectly lead to cancer progression. The mitogen-activated protein kinase (MAPK) signaling-mediated overexpression of miR-21 induces tumorigenesis- and metastasis-associated phenotypes, including cell migration and invasion in lung cancer [16], EGF-induced pancreatic cancer [17], and Her2/neu-overexpressing breast cancer [18]. Mir-21 expression is associated with cancer stem cell properties and is involved in stemness maintenance in multiple cancers, including pancreatic ductal adenocarcinoma cells [19,20,21,22]. In addition, miR-21 contributes to drug resistance in head and neck squamous cell carcinoma [23], breast cancer [24], acute myeloid leukemia [25], ovarian cancer [26], and colon adenocarcinoma [27]. The abundant expression of circulating miRNAs such as miR-629*, miR-660, and miR-141 were detected in the plasma of prostate cancer xenograft model system, leading to the potential use of circulating miRNAs as blood-based biomarkers of human cancer detection [28]. Furthermore, miRNA-based clinical therapy has made significant progress over the past decade. MiR-16-based nanoparticle-encapsulated microRNA mimics have been employed in phase I trials in recurrent malignant pleural mesothelioma patients [29]. Another prospective phase II clinical trial encompassing the stratification of chemo-refractory metastatic colorectal cancer patients with low miR-31-3p positively correlated with clinical benefits from anti-EGFR monoclonal antibodies [30]. Hence, in this special issue focusing on “MicroRNAs—Small Molecules with Great Potential in Tumorigenesis”, we intend to focus on a specific family of miRNAs that is indispensable for animal development and its deregulation among the pathogenesis of various diseases: the miR-200 family.

**Figure 2:**
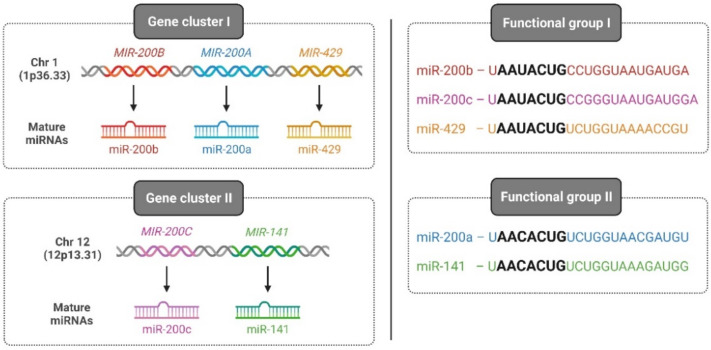
Classification of the human miR-200 family. Two categories of the human miR-200 family classification exist based on the chromosomal location of the gene (left—gene cluster I and II) and based on seed sequences that bind to 3′ UTR of cognate genes (right—functional group I and II). Illustration created with Biorender.com (https://biorender.com/, accessed on 1 June 2022).

Among vertebrates, the miR-200 family is one of the most-conserved miRNAs and consists of five members: miR-200a, miR-200b, miR-200c, miR-141, and miR-429. On one hand, based on its chromosomal location, the miR-200 family is divided into two clusters: cluster I, which is located on human chromosome 1 (1p36.33) encoding for miR-200a, -200b, and -429, and cluster II, which is located on chromosome 12 (12p13.31) encoding for miR-141 and -200c. On the other hand, based on the seed sequence that binds to target genes, the miR-200 family is classified into two groups: group I, comprising miR-200b, -200c, and -429 with a seed sequence: 5′-AAUACUG-3′, and group II, consisting of miR-200a and -141 with a seed sequence: 5′-AACACUG-3′ (Figure 2).

It is also speculated that members of the same miRNA family are segregated in two different locations on the genome, rendering miR-200s with more flexibility in imposing spatial, temporal, and tissue-specific control of target gene expression, since the expression of miR-200s is also subjected to direct regulation through histone modification [31,32]. Nevertheless, several exogenous stimuli and signaling cascades activate miR-200 members to mediate key cellular functions, which we will elaborate upon in the coming sections. Similarly, the deregulation of miR-200s is frequently observed in multiple pathological conditions such as tissue fibrosis, neurodegenerative diseases, and cancer, which are discussed in the later part of the article.

## 2. miR-200 Family in Development

The formation and establishment of two distinct layers of tissue, epithelium and mesenchyme, demarcate the basis of organ development and coordinated multicellularity in metazoans. The epithelium is characterized by a layer of cells adjacently linked through interconnecting cellular structures such as adherens junctions, tight junctions, desmosomes, and gap junctions. The obligate roles of each of these structures in epithelial cellular organization, adhesion, and selective permeability are extensively discussed in recent reviews [33,34,35,36]. In addition, epithelial cells display characteristic apical–basolateral cellular polarity (the apical pole facing the outer lumen and the basal pole facing the basement extracellular matrix), which is crucial for the asymmetric localization of proteins at distinct membrane domains and the orientation of microtubule networks during intra-cellular trafficking [37,38]. During early embryonic events such as embryonic implantation, gastrulation, and neural crest formation, a subset of epithelial cells from the epithelial layer of the embryo systematically loses its epithelial features through a process called epithelial-to-mesenchymal transition (EMT), thereby acquiring a mesenchymal phenotype. Unlike epithelial cells, mesenchymal cells show weakened cell–cell adhesion properties and lack apical–basal cellular polarity, resulting in cells with enhanced migratory behavior as well as the potential to degrade the underlying extracellular matrix. The salient bifurcation of these two contrasting cellular types during early embryogenesis is vital for successive vertebral column development and organogenesis [39]. Recently, studies focusing on the role of miRNAs during early embryogenesis have identified that the miR-200 family plays essential roles in the establishment and functioning of the epithelial phenotype during early embryogenesis and organogenesis that are detailed below, and a summary of specific miR-200 family members, their target genes, and the regulated function is listed in Table 1. 

In order to establish and safeguard epithelial cell identity during development, members of the miR-200 family deploy a multi-pronged approach, depending on the cellular context. In certain tissues, miR-200s function as potent repressors of the EMT process. For example, during neural crest cell migration, miR-200c and miR-145 target Sox-1 and Sox-9, respectively, to upregulate E-cadherin expression and suppress EMT [42]. miR-200s target ZEB1 during the differentiation of human embryonic stem cells into hepatocytes and target ZEB2 in order to promote the late steps of postnatal forebrain neurogenesis [46,47]. Similarly, miR-200s’ mediated repression of ZEB1, ZEB2, and components of the Indian hedgehog signaling such as PTCH1, GLI2, and GLI3 is crucial during the endometrial development of embryo implantation [48]. In addition to directly curbing EMT, miR-200s suppress EMT-associated stem cell and self-renewal properties, which are crucial for the development and maintenance of unspecialized cellular niches. By directly targeting the transcription of stem cell self-renewal factor BMI1, miR-200c overexpressing murine mammary cells, when injected into the mammary fat pad of female mice, displayed aberrant, disorganized clusters of cells with non-functional mammary duct formation, potentially initiating myoepithelial-cell rather than luminal-cell differentiation [43]. Similarly, in the mesenchymal derivative of the breast epithelial cell line D492, the expression of miR-200c-141 reversed the EMT phenotype, and the co-expression of miR-200c-141 and ΔNp63 (a transcription factor crucial for stem-cell maintenance) restored epithelial differentiation and branching morphogenesis [50].

Recently, a few studies have demonstrated the unique role of miR-200s during the development of mammalian skin, which encompasses skin and hair follicle development and hair morphogenesis. The quantitative miRNA sequencing analysis of mouse neonatal skin has identified that the miR-200 family is among the most abundantly expressed miRNAs during embryonic skin development [51]. Mouse embryonic stem cells with activated sonic hedgehog signaling decreased miR-200s and activated the nuclear expression of ZEB1/ZEB2, leading to enhanced migration and skin wound healing [52]. Additionally, using double-end sequencing of cashmere goats during fetal periods, the expression of miR-200s was upregulated in pregnancy samples from day 55 and 66 when compared to pregnancy samples from day 45, underlining the significance of miR-200s in hair follicle development [53].

The murine dental epithelial stem cell niche is solely responsible for maintaining the stem cell population through self-renewal, while the spatiotemporal elevated expression of transcription factors such as Sox2, Bmi1, and Pitx2 stimulate the differentiation of dental epithelial cells that make up the lower incisor [54,55]. Therefore, the maintenance and differentiation of the lower incisor epithelial population offers an ideal model system to investigate the underlying role of miR-200s in epithelial stem cell renewal and differentiation. Along this line, recent histological and RNA sequencing analyses of murine dental epithelial stem cells show that miR-200 expression is required for the differentiation of terminally located progenitor cells and ameloblasts, as well as during the maintenance of the epithelial dental stem cell niche [56,57,58]. Furthermore, embryos depleted of miR-200c show defects in oral epithelial tissue invagination, leading to a reduction in incisor tissue composition and length. Using a conditional knockout system of dental stem cells and ameloblasts (cells that generate enamel), Cao et al. have shown that Pitx2 directly activates the expression of the miR-200c/141 cluster and miR-203, which subsequently inhibits noggin expression and leads to increased BMP signaling activity, leading to epithelial cell differentiation [45]. In addition, another independent investigation has shown that elevated BMP signaling during the early stages of mouse somatic cell reprogramming induces the expression of miR-205 and miR-200 family members, to reinforce an MET phenotype. This reveals the presence of miR-200-mediated epithelial differentiation as being vital during the initiation phase of reprogramming [59]. Therefore, these findings illustrate that BMP signaling-mediated miR-200 expression is crucial during the maintenance of dental epithelial stem cells, as well as for the differentiation of progenitor cells.

While the significance of the miR-200 family during EMT suppression was initially recognized in cancers, investigations along the same period have shown that the miR-200 family indeed plays distinct roles during embryogenesis and development, which is not confined just to the establishment and maintenance of the epithelial phenotype. In this section, we elaborate upon the role of the miR-200 family in the neurosensory epithelium such as olfactory neurogenesis and taste sensory organs. In mammals, the vomeronasal organ (VNO) and the main olfactory epithelium (MOE) are made of pseudostratified epithelial cells and bipolar sensory neurons, which are essential for the detection of pheromones and volatile odorants, respectively [60]. In situ hybridization experiments in mouse embryonic MOE showed strong expressions of miR-200a, -b, and -429, detected as early as E9.5 (first distinguishable stage of olfactory development) and maintained stable expression until E13.5 [61]. Moreover, the expression of all miR-200 members was observed in immature and mature neuronal cell layers of MOE and VNO, highlighting the persistent role of miR-200s in adult MOE and VNO neurogenesis. Intriguingly, the intranasal delivery of CRISPR-Cas9-mediated miR-200b/a knockdown in the MOE caused a significant loss of differentiated olfactory sensory neurons, accompanied by a dramatic reduction in olfactory-mediated male–male aggressive behavior and male–female mating behavior, which is mechanistically mediated through miR-200/TET3/REST signaling [40]. Mutant mice null for *Dlx5*, a homeogene that controls olfactory receptor neuron differentiation, showed a reduced expression of miR-9 and miR-200s, and the subsequent knockdown of miR-9 and miR-200s in zebrafish embryos led to defective olfactory placode organization as well as the altered differentiation and migration of olfactory receptor neurons [49]. In addition to regulating olfactory epithelial differentiation, miR-200s are implicated in the development of other essential neurosensory organs. Zebrafish embryos carrying morpholinos (anti-sense oligonucleotide analogs that are used for generating knockdown embryos) targeted against miR-200 expression resulted in a reduction in taste bud cells, and the upstream activation of FGF and Notch signaling is essential for miR-200 activity [41]. Similarly, the characterization of miRNAs in the developing submandibular gland revealed that miR-200c specifically regulates FGFR-dependent epithelial end bud proliferation and branching morphogenesis [44]. Based on the abovementioned investigations, the function of miR-200s is evidently essential for the development and functioning of selected sensory organs.

Furthermore, miR-8, the insect homolog of miR-200, is regarded as a pleiotropic regulator of *Drosophila* development, including neuroepithelial expansion [62], steroid signaling-mediated body size regulation [63,64], and pigmentation patterning [65]. miR-8 activity is also essential during mosquito oogenesis, such as the proper secretion of yolk protein precursors like vitellogenin and lipophorin in developing oocytes, through the Wingless signaling pathway [66].

## 3. miR-200 Family in Pathophysiology

The aberrant expression of the miR-200 family is involved in a group of pathophysiologic conditions such as cancer, diabetes, asthma, autoimmune diseases, kidney diseases, and neurodegenerative diseases. In this section, we would like to elaborate upon the role of miR-200s on the pathophysiology of neurogenerative diseases and fibrosis.

### 3.1. miR-200 Family in Neurodegenerative Diseases

In general, disorders characterized by the progressive degeneration of the structure or function of the central nervous system (CNS) are collectively referred as neurodegenerative diseases. Representative neurodegenerative diseases include Alzheimer’s disease (AD), Parkinson’s disease (PD), amyotrophic lateral sclerosis (ALS), Huntington’s disease (HD), multiple sclerosis, and prion diseases. Consequently, they cause significant defects in motor and cognitive ability [67]. An eminent study of the miR-8 mutants has shown that these mutants displayed high levels of apoptosis in the brain, behavioral defects, and an impaired neuromuscular coordination phenotype in *Drosophila* legs, which prompted the potential role of miR-200s in neurodegenerative disorders in mammals [68]. Recently, a number of studies have revealed that members of the miR-200 family are differentially expressed in the human brain and, more importantly, modulate genes associated with specific neurodegenerative disorders (Table 2).

#### 3.1.1. miR-200 Family in Alzheimer’s Disease

Alzheimer’s disease (AD) is a progressive neurological disorder, characterized by an increasing level of memory loss and deterioration of cognitive functions that eventually lead to dementia [78]. In particular, the pathological landscapes of AD brains are well recognized by the deposition of beta amyloid peptide (Aβ) and the formation of intracellular neurofibrillary tangles (NFT) [79], and ER stress is believed to be the initial driver for neuronal cell loss in AD [80]. Several studies have demonstrated that members of the miR-200 family are found to be linked with AD progression. The overexpression of miR-200a in peripheral blood mononuclear cells from AD patients targets genes related to cell cycle and DNA repair [81]. Another report showed that the upregulation of miR-200a-3p targets SIRT1, an anti-apoptotic protein, in order to stimulate Aβ-induced neuronal apoptosis [70]. Similarly, miR-200c is overexpressed in the plasma of patients with moderate to severe forms of AD, indicating its association with the progression of the disease [78].

In turn, the decreased expression of miR-200b and -429 by Aβ42 leads to the expression of the amyloid precursor protein (APP). Increased APP levels, in turn, lead to an increase in Aβ42, which causes a further reduction of miR-200b. This generates a kind of vicious cycle that contributes to the progression of AD [71]. Furthermore, folic acid deficiency can also decrease the level of miR-200b. Thus, a folic acid-deficient diet stimulates APP overexpression and promotes Aβ generation [72,82]. Higaki et al. report that miR-200b/c reduces insulin resistance by targeting the ribosomal protein kinase S6 B1 [73]. This contributes to a reduction in the production of Aβ peptide and thus alleviates the Aβ-induced toxicity. On the contrary, the upregulation of miR-200c levels was observed in the serum of AD patients [78], denoting that the precise role of miR-200s in Aβ-induced ER stress remains to be clearly investigated. These findings highlight that the miR-200 family is an important player in the pathogenesis of AD and could be explored for the possibility of diagnostic markers of the disease.

#### 3.1.2. miR-200 Family in Parkinson’s Disease

Parkinson’s disease (PD) is the most common neurological movement disorder [83]. PD involves a progressive loss of neurons in the brain, especially dopamine-producing (“dopaminergic”) neurons in a specific area of the brain called the substantia nigra. The loss of more than 50% of DA neurons causes a corresponding reduction in the synthesis of dopamine neurotransmitters. This, in turn, is manifested by motor dysfunction and clinical symptoms such as resting tremor, slowness of movement, speech changes, and impaired posture and balance [84]. Moreover, the neurons of PD patients are enriched in the aggregated protein α-synuclein (α-syn). This causes impairment in pathways such as vesicle trafficking or activating neuroinflammation disorders [85]. Although the exact molecular mechanisms of PD are still unknown, many studies suggest a key role of the miR-200 family in the pathogenesis of Parkinson’s disease.

The point mutations in human α-syn (A53T) and transgenic murine model of α-synucleinopathy (M83 SCNA∗A53T) have been directly implicated in the onset of familial early PD [86,87]. The miRNA profiling of transgenic mice and the cerebrospinal fluid of PD patients showed a significant enrichment of miR-200a-3p expressions, denoting their potential role in PD pathogenesis [88]. Moreover, miR-200 expression was correlated with the severity of PD, as confirmed by high Hoehn and Yahr (H&Y) scores [89]. These results may indicate that miR-200a expression may be correlated with the severity of PD, and miR-200a may be an effective marker of PD disease progression. In addition, sirtuin (SIRT1), a histone deacetylases family of proteins, have a protective role in PD through the amelioration of oxidative stress-induced neural cell death and the suppression of α-syn-induced aggregate formation [90]. SIRT1 has also been shown to render anti-apoptotic functions by suppressing p53 activity through deacetylation and promote cell survival [91,92,93]. In the context of the miR-200 family, miR-200a also has the ability to induce SIRT1 downregulation and trigger the apoptosis of dopaminergic neurons, consequently contributing to the development of PD [74,94]. Interestingly, Delavar et al. [75] showed that miR-141 also targets SIRT1 expression and correlates with PD-related pathogenic processes. Mechanistically, in a 1-methyl-4-phenylpyridinium- (MPP+-) induced in vitro PD model, the upregulation of miR-141-3p induced increased apoptosis, oxidative stress, and mitochondrial membrane potential through the direct targeting of SIRT1 expression [69]. The same study also shows that resveratrol (a SIRT1 activator) blocked and sirtinol (a SIRT1 inhibitor) reversed the abovementioned biological effects of miR-141-3p, respectively. Therefore, these studies highlight that the miR-200 family has potential roles in the onset and progression of PD.

#### 3.1.3. miR-200 Family in Amyotrophic Lateral Sclerosis

Amyotrophic lateral sclerosis (ALS) is another progressive neurodegenerative disease that primarily affects motor neurons controlling voluntary muscles, and the resulting loss of these motor neurons leads to the deterioration of the coordinated muscle movements involved in walking, talking, eating, and, eventually, breathing [95]. Although the origins of ALS are sporadic in nature, a small number of cases are associated with genetic changes [96]. Over the years, about 20 genes have been associated with familial ALS, and many of these genes encode RNA-binding proteins, including Fused in sarcoma (FUS), a DNA/RNA-binding protein [97]. miR-141 is shown to regulate the expression of FUS, EWS, and TAF15 in differentiating neuronal cells, denoting that miR-141-mediated FUS regulation is observed during neurogenesis [76]. Interestingly, miR-141/200a and FUS are linked by a feed-forward regulatory loop where the prevalent FUS mutation in ALS masks miR-141/200a binding sites and contributes to the excessive accumulation of the FUS protein, eventually augmenting ALS pathogenesis [95].

In addition, miR-200c is directly regulated by FUS, which contributes to gene silencing. In turn, mutations in FUS reduce the silencing of genes targeted by miR-200c, which may be one of mechanisms involved in the development of ALS [77]. Additionally, Zhou et al. [98] found that the expression level of miR-200a was increased in the early stage and decreased in the later stage in ALS transgenic mice, indicating this microRNA as a potential marker for detecting the progression of ALS.

#### 3.1.4. miR-200 Family in Multiple Sclerosis and Prion Disease

Multiple sclerosis (MS) is the most common demyelinating disease of the CNS. It is considered an autoimmune disease in which the body’s immune system attacks its own tissues [99]. The epidemiology of multiple sclerosis in developing countries shows that there has been a sharp increase in the incidence of patients, with an overall incidence of 85.8 per 100,000 [100]. The cause of MS is unknown, but it appears that a combination of environmental factors, epigenetics, and genetics lead to ongoing immune attacks on the CNS [101]. Two preliminary assessments on MS patient samples showed that miR-141 and -200a levels were increased in the relapsing phase of MS patients compared to the remission and control groups [102,103]. In addition, elevated miR levels induce the differentiation of Th17 cells that are involved in the development of MS [102]. Prion disease is a rare, fatal, neurodegenerative disease caused by abnormally folded prion proteins in brain (PrP) [104]. Most cases of prion disease in humans arise spontaneously and their signs and symptoms typically begin in adulthood and worsen with time [105]. A pilot study has potentially revealed a correlation between prion disease and miR-200 family expression, where decreased levels of all members of the miR-200 family correlated with morphological changes of dendritic spines and synaptic dysfunction [106]. Although the differential expressions of the miR-200 family members are beginning to be documented in such neurodegenerative conditions, specific roles of these miRNAs in their etiology remain largely unknown.

### 3.2. miR-200 Family in Fibrosis

Fibrosis is one of the major pathological processes that affect vital organs such as the kidneys, liver, lungs, and intestines. It is characterized by impaired epithelial architecture and excessive deposition of extracellular matrix and fibrous connective tissue, which generates multiple inflamed scar tissues within the organ, eventually leading to organ dysfunction and failure. Furthermore, patients diagnosed with cystic fibrosis pose increased risk towards cancer progression, denoting that fibrosis might serve as a gateway to life-threatening illnesses [107]. At the molecular level, several studies have validated the activation of EMT during the early stages of fibrosis and is referred to as type 2 EMT [108,109]. Accordingly, with EMT-inhibiting roles, miR-200s have been also implicated during tissue fibrosis.

Using a unilateral ureter obstruction model, Oba et al. have shown that the injection of 0.5 nM of pre-miR-200b (precursor) efficiently inhibited the rise of collagen and fibronectin levels in obstructed kidneys, and could ameliorate renal tubulointerstitial fibrosis [110]. Similarly, the collection duct-specific inhibition of miR-200 activity in a transgenic mouse model evoked the expression of profibrotic target genes and inflammatory cytokines such as Mcp1, Il6, and Cxcl2 [111]. Furthermore, the downregulation of miR-200 members in renal fibrosis is primarily mediated through TGF-β1, and accordingly, the overexpression of miR-141 or -200b hindered Smad-dependent TGF-β signaling and downstream EMT phenotypes [112,113,114].

Levels of miR-200a and miR-200c were also significantly downregulated in murine lungs in a bleomycin-mediated fibrosis model as well as in patients with idiopathic pulmonary fibrosis (IPF), and the restoration of miR-200 expression in senescent IPF cells resumed normal regenerative functions [115,116,117]. In the case of liver fibrosis, miR-200s seem to play a pro-fibrotic role. In a CCL4-induced liver fibrosis model, miR-199 and miR-200 levels were significantly upregulated in comparison to the control groups [118]. Accordingly, the expression of miR-200s were elevated in serums of patients diagnosed with non-alcoholic fatty liver disease (NAFLD), and NAFLD mice treated with miR-200 inhibitor ameliorated liver fibrosis. Mechanistically, GRHL2 was overexpressed in the serum of NAFLD patients, which in turn negatively targeted SIRT1 and also activated miR-200 and the MAPK signaling pathway, aggravating liver fibrosis and intestinal mucosal barrier dysfunction [119]. GRHL2 is regarded as a gatekeeper of epithelial phenotype and differentiation, also involved in the direct activation of the miR-200 family [120].

### 3.3. miR-200 Family in Cancer

**Figure 3:**
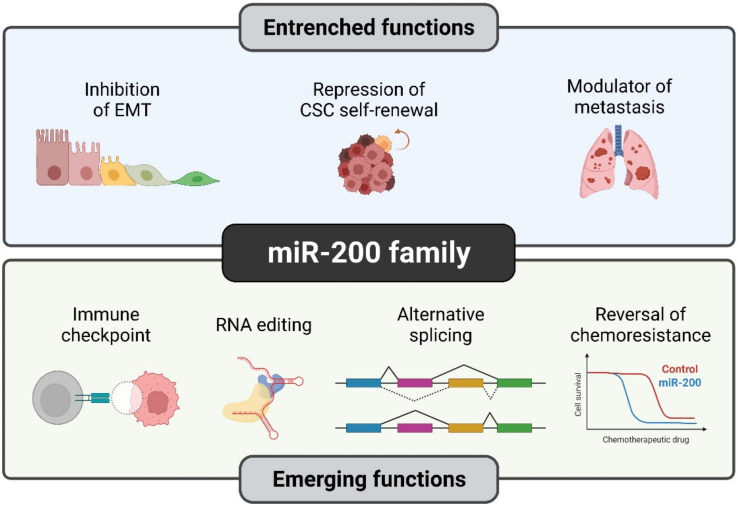
Established and novel functions of the miR-200 family in human cancers. Although certain functions of miR-200s such as EMT suppression are quite well understood, members of the miR-200 family have recently been studied in detail for their novel roles such as RNA editing and reversal of chemoresistance. Illustration created with Biorender.com (https://biorender.com/, accessed on 1 June 2022).

Over the past two decades, the role of miR-200s in cancer has been extensively studied, which has led us to distinguish the established (well-explored) and emerging functions that are exclusively observed during cancer progression (Figure 3).

The dysregulation of miR-200 expression in various entities of human cancer (reviewed in [121]) assigns it with tumor-promoting as well as tumor-suppressive effects. Through the direct targeting of genes that are involved in tumor-promoting mechanisms, members of the miR-200 family, individually or collectively, have been reported to be involved in the regulation of EMT/MET and metastasis [122,123], in the regulation of the cell cycle and apoptosis [74,124], as well as its deregulation during chemoresistance [125], cancer stemness [43,126], and, more recently, also in modulating intra-tumoral immune cell function [127,128,129]

#### 3.3.1. miR-200 Family in Cancer-Associated EMT and MET

In the metastatic cascade, miR-200 exerts a context-dependent role. In general, the expression of miR-200 family members in the primary tumor strengthens an epithelial phenotype, thereby preventing EMT. Members of the miR-200 family primarily block EMT through direct repression of the EMT inducers ZEB1 and ZEB2, and impede tumor cell dissociation, migration, and invasion, which potentially combats the initial stages of the metastatic cascade, thus implicating the miRNAs’ tumor-suppressive effect. During the late 2000s, five research groups independently validated that miR-200s strongly repress EMT in multiple cancer types by directly targeting the major EMT transcription factors ZEB1 and ZEB2 [130,131,132,133,134]. The 3′UTR of ZEB1 and ZEB2 contain eight and nine miR-200 binding sites, respectively, which results in a strong and effective repression of the ZEB1/2 transcripts [132,134]. ZEB1 and ZEB2, on the other hand, suppress the transcription of all members of the miR-200 family [131,135]. These data illustrate that ZEB1/2 and miR-200s are not just functional rivals during cellular differentiation (EMT) and dedifferentiation (MET) processes in cancer, but mutually control the expression of each other, generating a double-negative feedback loop [136].

miR-200s directly target members of the Notch signaling pathway such as Jag1, Jag2, Maml2, and Maml3 to suppress pancreatic and lung adenocarcinoma proliferation and metastasis [137,138,139]. Several studies have reported that miR-200 family members influence cancer cell invasion by regulating the proteins involved in remodeling of the cytoskeleton. This interaction may be dependent on [140] or independent of the ZEB1/miR-200 axis [141,142].

Although cancer-associated EMT is regarded as a binary process driving cellular epithelial identity towards a mesenchymal phenotype, meticulous studies in the recent decade have acknowledged that this process is executed in a rather gradual mode, with the presence of one or more intermediary cellular states, each exhibiting distinct phenotypical, transcriptional, and epigenetic signatures [143,144,145]. Cells harboring such intermediate or hybrid EMT characteristics are prevalent across multiple cancer types such as breast [146,147], prostate [148], ovarian [143], and non-small lung cancer [149]. Moreover, cells with hybrid EMT state display increased tumorigenicity [150] and metastatic potential [145,151]. Subsequent mathematical and computational studies have highlighted that the core EMT decision-making circuit consists of two dynamically interconnected inhibitory feedback loops: one between miR-200 and ZEB1/2 [137], and the other between miR-34 and SNAIL1/2 [152]. Such regulatory feedback mechanisms are crucial during the generation and maintenance of hybrid EMT cellular states [153,154,155,156]. Recently, Garinet et al. profiled 176 resected non-small cell lung carcinoma specimens and identified that most tumors were associated with an EMT-hybrid state and miR-200s expression profiles derived from those samples were utilized for the identification of good prognostic groups that are unrelated to conventional EMT scores [157]. Furthermore, a recent TGF-β1-induced single-cell RNA sequencing study re-established that the gradual deregulation of miR-200 family expression is observed during intermediate EMT cellular clusters [158]. All the above studies denote that cancer-associated EMT is a dynamic, continual process instead of a terminal differentiation program and that sequential deregulation of the miR-200 family is observed during the generation of intermediate EMT cellular states.

#### 3.3.2. miR-200 Family’s Tumor-Promoting Roles during Tumorigenesis and Metastasis

Although numerous studies have elucidated the indispensable role of the miR-200 family in cancer metastasis-associated functions such as tumor cell dissemination, invasion and angiogenesis, miR-200s are active players during the early stages of tumorigenesis such as neoplastic transformation. MiR-200s seem to render a pro-tumorigenic factor in many cancers, which is in contrast to their tumor-suppressive roles in later stages of the metastatic process. Xenografts carrying miR-141 or miR-200a overexpressing Kras-transformed ovarian fibroblasts formed faster and larger tumors by inhibiting p38α and stimulating the oxidative stress response [159]. In the case of ovarian carcinoma, a conditional mouse model showed that the loss of Dicer, a miRNA biogenesis factor, promoted the epithelialization of fallopian tube stromal cells and initiated tumorigenesis [160]. Accordingly, elevated expressions of miR-200 and E-cadherin were identified in human and white Leghorn hen ovarian cancer tissues, highlighting their roles in the initial development of ovarian carcinoma [161,162]. Ovarian cancer cells with miR-200 knockdown when grown on a three-dimensional (3D) organotypic setting showed an increased number of lumina, which is due to mitotic spindle disorientation, loss of cellular polarity, and a collective migration phenotype, facilitating ovarian carcinogenesis [163]. At the molecular level, the loss of collective migration in these 3D structures is due to the disruption of ROCK-mediated myosin-II phosphorylation and SRC signaling. The increased expression of miR-200 in early-stage lung adenocarcinomas in vitro and in vivo activated PI3K/AKT signaling through FOG2 targeting and promoted tumor-initiating spheroid growth formation [164]. Early ductal carcinomas in situ (DCIS) lesions of the breast tissue are non-obligatory precursors of invasive breast cancer, yet their severe microenvironment with hypoxia, nutrient deprivation, and acidosis facilitate tumorigenesis [165,166]. Subtype-specific miRNA profiling of normal, DCIS, and invasive breast carcinoma cohorts showed significant enrichment of miR-21-5p and all members of the miR-200 family in the DCIS samples, denoting their potential tumorigenic role in breast carcinogenesis [167].

In the context of cancer progression and metastasis, it is important to note that miR-200s support an MET of mesenchymal tumor cells at distant organ sites, thereby promoting metastatic colonization. In breast cancer, miR-200s promote the metastatic colonization of secondary organs by targeting the cancer cell secretome, subsequently influencing the tumor microenvironment [168,169]. In addition, miR-200c, miR-192, and miR-17 were identified as targeting genes involved in extracellular matrix remodeling. The expression of the three miRNAs in tumor-associated fibroblasts may therefore suppress the invasion of colorectal cancer cells [170]. Further studies on miR-200 in the tumor microenvironment recently revealed that apoptotic MCF7 breast cancer cells release miR-200c, which is taken up by tumor-associated macrophages. This leads to the overexpression of miR-200c in the tumor-associated macrophages, followed by the reduced expression of a set of pro-migratory miR-200c targets and a reduced capacity of the macrophages to infiltrate into tumor spheroids [129]. Taken together, these findings show that miR-200 is a crucial player in the crosstalk between tumor cells and tumor-associated stromal and immune cells. This crosstalk may modulate the cells’ invasive behavior with tumor-promoting or suppressing effects, depending on the releasing and receiving cell. This points to the fact that the role of miR-200s in tumor progression extends beyond the regulation of transition between EMT and MET and the ZEB/miR-200 feedback loop.

#### 3.3.3. miR200 Family in Radiotherapy

Radiotherapy still remains one of the main front-line treatment options in several cancer types including breast, lung, prostate, cervical, and head and neck cancers. The response of cancer cells towards radiotherapy is mostly affected by the tumor size. However, recent preclinical studies have highlighted that miRNAs, especially miR-200s, contribute towards radiotherapy response in multiple cancer types [171]. In breast cancer cells, it has been shown that high miR-200c expression is important for radiosensitivity, while decreased expression is associated with radioresistance [172]. This has been demonstrated in studies with cell lines showing different basal levels of miR-200c expression. The MCF-7 cell line, which has a high level of miR-200c expression, showed a higher sensitivity to radiotherapy compared to the MDA-MB-231 cell line. Additionally, higher expression of miR-200c increases the sensitivity to radiation by inhibiting cell proliferation and by increasing apoptosis and DNA double-strand breaks [173]. Further studies showed that the sensitization of cancer cells to radiation therapy with high levels of miR-200c is mediated by targeting TBK1 and VEGF-VEGFR2, resulting in cell apoptosis [174]. Moreover, Sun et al. identified ubiquilin 1 (UBQLN1) as a functional target of miR-200c, where miR-200c sensitizes breast cancer cells to radiation in a manner associated with the inhibition of radiation-induced autophagy [174]. In turn, Wang et al. speculated that radiation resistance is promoted by the interaction of the long coding RNA, LINC02582, with USP7 to deubiquitinate and stabilize CHK1. Therefore, they proposed that the miR-200c/LINC02582/USP7/CHK1 signaling axis could be a therapeutic target to improve the breast cancer response to radiotherapy [175].

In addition, miR-200c plays a role in the effectiveness of radiotherapy in lung cancer. The high expression of miR-200c increases radiation sensitivity by regulating the oxidative stress response by targeting PRDX2, GAPB/Nrf2, and SESN1 and by inhibiting the repair of radiation-induced double-strand breaks [176]. Next, miR-200a affects the radiosensitivity of non-small cell lung cancer (NSCLC) cells. The analysis of clinical data in NSCLC patients showed that miR-200a negatively regulates HGF expression, resulting in reduced cancer cell invasion and metastasis. Moreover, the miR-200a/HGF pathway also influences the radiosensitivity of NSCLC cells. This was confirmed in experiments on cell lines A549 and H1299, where the overexpression of miR-200a after irradiation resulted in the increased apoptosis and DNA double-stranded breaks of these cancer cells [177].

Interestingly, miR-200a is detected in the saliva of patients with head and neck squamous cell carcinoma after radiation treatment. The analysis of the distribution of miR-200a expression during and after radiotherapy showed that the miR level is significantly higher 12 months after treatment compared with the baseline state [178]. It may constitute a potential tool in monitoring the response to radiotherapy in patients with HNSCC. Along this line, radiation-induced oral mucositis (RIOM) is one of the more prevalent side effects of radiotherapy in HNSCC patients. A preliminary study identified that all members of the miR-200 family were significantly upregulated in RIOM formation, and the knockdown of miR-200c led to a reduction of proinflammatory cytokine synthesis and reactive oxygen species generation [179].

In addition to all of the abovementioned studies, two new reports this year implicate the association of miR-200s with radiosensitivity in other cancer types. The suppression of miR-200b/c in esophageal squamous cell carcinoma impaired with cell sensitivity to concurrent chemoradiotherapy treatment with or without surgery [180]. Similarly, tumor xenografts carrying miR-200a/b/-429 overexpressing cervical carcinoma cell lines were significantly sensitized to radiotherapy, which is also independent of tumor hypoxia [181]. Therefore, the prospective role of the miR-200 family as a predictor of response to cancer radiotherapy has been revealed in work over the last decade and certainly demands more research.

#### 3.3.4. miR-200 Family in Chemotherapy

Dealing with the emergence of therapy resistance poses a major challenge in the treatment of cancer patients. Recent literature has reported that miR-200s are involved in modulating the response of cancer cells to anti-cancer therapy by influencing the status of certain adenocarcinomas towards chemo- and radiosensitivity. Importantly, therapy resistance occurs through numerous resistance mechanisms such as increased DNA repair capacity, elevated drug efflux through ATP-binding cassette (ABC) transporters, varying expression of β-tubulin isotypes, changes in cell cycle, inhibition of apoptosis, and response to oxidative stress [182]. The participation of miR-200 in these processes has been well described, thereby miR-200s may enhance the therapeutic possibilities of EMT-associated cancer metastasis and may even predict therapeutic response [140,183,184]. Crudele et al. reviewed the miR-200 family network’s connection with resistance to six different anti-cancer treatments [185]. In this regard, the restoration of miR-200c expression in ovarian and endometrial cancer cells leads to increased sensitivity to microtubule-binding chemotherapeutic agents through the reduction of class III β-tubulin (TUBB3) [125]. Similarly, in chemoresistant pancreatic cells, the induction of miR-200s and miR-203 expression through the class I HDAC inhibitor mocetinostat restored drug sensitivity to standard chemotherapeutics such as gemcitabine and docetaxel [186]. The oxaliplatin-resistant colorectal cancer cell line showed increased expression of SUZ12, a Polycomb-repressive complex 2 subunit, and decreased expression of the miR-200 family [187]. In a similar fashion, the expression of miR-200c alone enhanced the chemosensitivity and reduced the metastatic potential of p53null claudin-low breast cancer mouse models [188]. The depletion of miR-200c significantly reduced 5-flurouracil-induced apoptosis and caspase 3 activity in colorectal HCT-116 cells [189], and the inhibition of miR-141/200c in the ovarian cancer cell line OVCAR-3 led to resistance to paclitaxel and carboplatin [190]. The overexpression of miR-200c in melanoma cells decreased resistance to cisplatin as well as a BRAF- and a MEK-inhibitor through the downregulation of the ABC transporters ABCG2, ABCG5, and MDR1 directly or indirectly through the downregulation of BMI-1 [183,191]. Intriguingly, a recent pilot study has shown that the anticancer drug cisplatin interacts with pre-miR-200b and decreases the production of mature miR-200b expression in ovarian cancer cells [184]. Therefore, a direct correlation between miR-200s and a drug sensitivity phenotype has been established primarily using adenocarcinomas; however, the underlying mechanistic insights are still being delineated.

It is important to mention that there are also reports that observe the opposite effects, and describe miR-200 as conferring chemoresistance. In a study by Yu et al., the expression of miR-200a correlated with poor response to preoperative chemotherapy and poor prognosis in breast cancer patients. Additionally, miR-200a was described to confer chemoresistance to gemcitabine by targeting TP53INP1 and YAP1 [192]. *TP53INP1* is a p73 target gene that inhibits cell growth and promotes cell death, and is induced by stress in response to DNA damage, i.e., by cisplatin [193]. In hepatocellular carcinoma, the overexpression of miR-200a-3p increased resistance to 5-FU by targeting DUSP6. In this setting, the inhibition of miR-200a-3p decreased cell growth and viability after 5-FU treatment, thereby sensitizing Hep3B cells to different anti-cancer drugs, including 5-FU, cisplatin, and doxorubicin [194].

The administration of anticancer drugs or radiotherapy is proposed to lead to an accumulation of reactive oxygen species (ROS). These ROS interfere with different cellular processes such as cell survival and motility, and the oxidative stress influences miRNA expression patterns. The miR-200 family was described to be involved in the oxidative stress response by regulating KEAP1 expression in breast and ovarian cancer [149,150,179]. The introduction of miR-141/-200c into a paclitaxel-resistant ovarian cell line confers resistance to carboplatin, while altering the expression of genes involved in balancing oxidative stress [190].

In summary, miR-200 family members target a plethora of genes involved in fate determining processes within cancer cells as well as in cells of the tumor microenvironment. A selection of these miR-200 target genes is compiled in Table 3. Seemingly contradictory effects may occur, which require a precise analysis of cellular context and spatiotemporal expression pattern of the miR-200s in order to predict the outcome of a potential therapeutic interference. The fact that cells may release miRNAs assigns miR-200s a role in inter cellular communication and makes them detectable in body fluids for a potential use as biomarkers which is discussed in the following section.

## 4. miR-200 Family as Predictive Markers

The miR-200 family has substantial influence on cancer cell motility and invasive behavior through the direct suppression of EMT and metastasis formation in different tumor types. Consequently, many studies have suggested that the miR-200 family has great potential to be key biomarkers in a variety of diseases, including cancer (recently reviewed in [199], Table 4) and neurodegenerative diseases [200,201]. For instance, a recent meta-analysis study encompassing 24 eligible breast cancer articles and 16,565 subjects showed a significant correlation between high miR-200s expression and the poor overall survival of breast cancer patients, while the downregulation of miR-200s was associated with the poor survival of triple-negative and luminal breast cancer patients [202]. Another meta-analysis study involving eight studies with data from 1150 bladder cancer patients showed that the high expression of the miR-200 family was associated with better overall survival and relapse-free survival, which is regarded as a reliable prognostic biomarker in bladder cancer patients [203]. Likewise, the low expression of miR-200c in breast cancer was correlated with a poor response to neoadjuvant chemotherapy [200]. Furthermore, the profiling of candidate miRNAs in plasma samples from metastatic breast cancer patients showed that circulating tumor cells enrich miR-200s, miR-203, and miR-375 expressions and are associated with the onset of metastatic disease, further highlighting their prognostic significance [204,205]. A similar trend of higher levels of circulating miR-200s expression in plasma and exosomes of colorectal cancer patients were correlated with poor prognosis [206,207]. The overexpression of miR-200b and -200c was observed in the serum exosomes of pancreatic ductal adenocarcinoma patients, in addition to a correlation between their expression and shorter overall survival [208]. Furthermore, elevated expressions of miR-141 and -200c in non-small cell lung cancer and colorectal cancer were associated with poor prognosis and shorter overall survival [209,210].

In addition to the potential utility of miR-200s in solid carcinoma diagnosis/prognosis, certain studies have highlighted the prospect of miR-200s as diagnostic markers in liquid biopsies. Zuberi et al. identified a direct correlation of miR-200a/c expression in the serum of epithelial ovarian cancer patients in advanced stages of disease (stages III and IV) when compared with early stages (I and II) [216]. In this study, the authors demonstrate a correlation between increased miR200a/c serum levels, which are associated with an aggressive tumor progression, combined with poor prognosis in ovarian cancer. Similarly, miR-21, miR-200s, and miR-205 were among the abundant RNA biomarkers detected in the biofluids of ovarian cancer patients when compared to controls [223]. The RNA profiling of exosomes isolated from serum, plasma, or pleural effusions of patients with lung cancer [224], cholangiocarcinoma [222], colon cancer [207], and pancreatic ductal adenocarcinomas [208] showed the miR-200 family as being one of the top differentially expressed microRNAs in distinguishing those diagnosed with benign disease. Taken together, the differential expression of miR-200s during varying stages of cancer progression and their distinctive expression in different cancer types render a prognostic and/or predictive value in multiple tumor types.

## 5. Conclusions and Future Perspectives

In spite of being single-stranded, non-coding oligonucleotides and encoded by less than 1% of the human genome, miRNAs are known to regulate over 60% of human protein-coding genes [225]. Therefore, it is imperative to claim that miRNAs are one of the major gene-regulatory elements, which exert control on almost every aspect of cellular events such as growth, differentiation, homeostasis, and death. It is also apparent that the deregulation of miRNAs is directly associated with the onset and progression of multiple diseases and pathological conditions. Furthermore, recent reviews have determined that miRNAs have roles in newer avenues, including drug addiction [226], neonatal sepsis [227], and the alteration of synaptic plasticity in depression [228,229], illustrating that this class of ncRNAs displays versatile functions in several aspects of cellular development as well as in pathological conditions.

Among the most versatile miRNAs, one or multiple members of the miR-200 family are directly involved in regulating almost all of the abovementioned aspects of cellular events, which we have comprehensively elaborated in the earlier sections. The initial function of miR-200s begin as early as the migration of neural crest cells and are crucial players in epithelial differentiation and branching morphogenesis. In particular, miR-200-mediated gene expression regulation is essential during mammalian skin, hair follicle, dental, and sensory organ development. Interestingly, recent studies are beginning to understand the miR-200s’ role in stem cell/progenitor cell populations [58,230], denoting that the role of miR-200s in embryonic and organ development is still underexplored.

In terms of the role of miR-200s in cancer, five pioneering yet independent publications in 2008 [130,131,132,133,134] revealing the EMT- and metastasis-suppressing roles of miR-200s in multiple cancer entities not only cemented an indispensable spot for the miR-200 family in comprehending cancer progression, but also prompted the scientific community to contemplate and investigate miR-200s and other miRNAs from a pathological perspective. Since then, an incessant surge in the number of publications identifying the association between miR-200s expressions and the pathogenesis of several human ailments has been observed. In this review, we have reviewed both the established functions of miR-200s, such as EMT suppression and cancer stemness inhibition, as well as newly emerging functions, such as RNA editing, alternative splicing, and the reversal of chemoresistance. Such new emerging functions underline the fact that miR-200s are not mere deregulators of oncogenes, but possess diverse potentials in combating and intervening in cancer metastasis. In contrast to the suppressive roles of miR-200s listed here, researchers have also found that members of the miR-200 family impose pro-tumorigenic functions, such as during tumor initiation and metastasis progression. This paradoxical phenomenon is not unique to the miR-200 family, serving as a reminder that these molecules execute functions based on the cellular context and upstream signaling events. Furthermore, the expression and, thereby, the functionality of miR-200s are often directly regulated or deregulated by transcription factors that are on the other side of the functional spectrum. Recent studies on three-dimensional (3D) chromatin interaction studies at the genomic level have speculated that local chromatin conformational changes at the 3D genome levels influence the expression of ncRNAs, including miRNAs [231,232]. Therefore, genome-wide chromatin conformation studies on the miR-200 locus across multiple pathological situations (chromatin conformational changes of cancer cells on miR-200 loci before and after EMT, drug sensitivity, etc.) could expose novel mechanisms through which miR-200-mediated disease progression or repression could be achieved.

Lastly, several studies and systemic reviews have already highlighted the prospective functions of miR-200s as a prognostic biomarker. However, efforts to utilize miR-200s and other related ncRNAs as commercial biomarker test kits are still lacking. On the other hand, simple and inexpensive commercial test kits are now available for quantifying other genetic abnormalities such as *TERT* promoter mutations [233], and the number of CGG repeats in the fragile X mental retardation 1 (*FMR1*) gene in Fragile X syndrome (FXS) [234]. Such initiatives in the coming years are essential for the successful utilization of miRNA-based biomarkers in the preclinical and clinical settings.

## Figures and Tables

**Table 1:** miR-200s, target genes, and their roles in development.

miRNA	Target Gene	Function in Development	References
miR-200a/-200b	TET3	Olfactory-mediated behaviors and globose basal cell proliferation and differentiation in the mouse main olfactory epithelium (MOE)	[40]
miR-200a/-200b/-429	Sox2	Taste bud formation	[41]
miR-200c	Sox-1	Neural crest cell migration	[42]
	BMI	Regulating self-renewal and differentiation of stem cells	[43]
	Vldlr	FGFR-mediated epithelial end bud proliferation during branching morphogenesis	[44]
miR-141/-200c	noggin	Epithelial cell differentiation and tooth development	[45]
All members of the miR-200 family	ZEB1	Differentiation of human embryonic stem cells into hepatocytes	[46]
	ZEB2	Promote late steps of postnatal forebrain neurogenesis	[47]
	ZEB1, ZEB2, PTCH/GLI	Endometrial development of embryo implantation	[48]
	Foxg1	Olfactory receptor neuron differentiation, extension and connectivity of the olfactory axons, migration of the GnRH neurons	[49]

**Table 2:** miR-200s, target genes and their roles in neurodegenerative diseases.

Disease	miRNA	Target Gene	Function in Neurodegenerative Diseases	References
Alzheimer’s disease	miR-141	SIRT1	Promote Aβ-induced neuronal apoptosis	[69]
	miR-200a			[70]
	miR-200b/-429	APP	High expression of APP correlating with accelerated accumulation of the Aβ in brain and take part in the progression of AD	[71,72]
	miR-200b/c	S6K1	Reduction in Aβ secretion and/or Aβ-induced spatial memory impairment by promoting activation of the insulin signaling pathway	[73]
Parkinson’s disease	miR-200a	SIRT	Involved in DA neurons cell death via P53 and FOXO signaling pathways as a possible reason for PD pathogenesis	[74]
	miR-141		Induce neuronal apoptosis and oxidative stress	[75]
Amyotrophic lateral sclerosis	miR-141	FUS, EWS, TAF15	Involved in the differentiation of neuronal cells	[76]
	miR-200c	FUS	Promote miR-200c-mediated gene silencing	[77]

**Table 3:** Summary of miR-200s, target genes, and intervening mechanism in multiple cancer types.

miRNA	Target Gene	Mechanism Affected	Result	Cancer Types	References
miR-141, -200a	p38α	Response to oxidative stress	Paclitaxel sensitivity	Ovarian cancer	[159,195]
miR-200a	DUSP6	ERK signaling	Promotes drug resistance to 5-FU, doxorubicin, and cisplatin	Hepatocellular carcinoma	[194]
	TP53INP1	Cell cycle arrest and apoptosis	Resistance to chemotherapy	Breast cancer	[192,193]
	YAP1	Hippo signaling pathway; cell proliferation and suppression of apoptosis	Resistance to chemotherapy	Breast cancer	[192,196]
miR-200b	MEOSIN	Organization of cytoskeleton (actin filaments)	Remodeling of cytoskeleton independent of ZEB1/miR-200 axis through a moesin-dependent pathway	Breast cancer	[141]
miR-200c	BMI1	Regulation of cell cycle, stem cell self-renewal	Alteration of stem cell functionality	Breast cancer	[43]
	FHOD1/PPM1F	Organization of cytoskeleton (actin filaments)	Remodeling of cytoskeleton independent of ZEB1/miR-200 axis. Regulation of stress fiber formation; repression of migration and invasion.		[142]
	MYLK, TKS5		Remodeling of cytoskeleton dependent of ZEB1/miR-200 axis. Invasive potential, formation of invadopodia.		[140]
	TUBB3	Organization of cytoskeleton (microtubuli)	Increased sensitivity to microtubule-binding chemotherapeutic agents (paclitaxel and others)	Ovarian cancer, endometrial cancer	[125]
miR-200s	FOG2	PI3K/AKT pathway	Survival and proliferation	Lung cancer	[195]
	Jag1, Jag2, Maml2, Maml3	Notch signaling pathway	Suppression of cell proliferation and metastasis	Pancreatic and lung adenocarcinoma and basal type of breast cancer	[137,138,139]
	KEAP1	Keap1/Nrf2 signaling pathway	Oxidative stress response	Breast cancer and ovarian cancer	[197,198]
	SEC23A	Cancer cell secretome	Targeting secretion of metastasis-suppressive proteins; influencing tumor microenvironment; promoting metastatic colonization	Breast cancer	[168,169]
	ZEB1 and ZEB2	EMT inducing transcription factors; repression of E-Cadherin	Repression of EMT	Non-small cell lung cancer and breast cancer	[130]
				Pancreatic cancer, colorectal cancer, and breast cancer	[131]
				Breast cancer	[132,133]
				NCI60 panel of cancer cell lines	[134]

**Table 4:** A summary of differential changes in miR-200s expression as predictive biomarkers of multiple cancer types. The type of sample used and method of detection are also included.

Sample Used	Method of Detection	miRNA Detected	Pattern of Expression	Cancer	References
Ascitic fluid	RT-qPCR	All members of the miR-200 family	Upregulated	Ovarian cancer	[211]
Serum/plasma	RT-qPCR	All members of the miR-200 family	Upregulated	Ovarian cancer	[212]
		miR-200a, -200b & -200c			[213,214,215,216,217]
		miR-200c			[218]
Serum	RT-qPCR	miR-200c	Upregulated	Colorectal cancer	[219]
Serum exosomes	Microarray	miR-200b	Upregulated	Ovarian cancer	[220]
	RT-qPCR	miR-200c			[221]
Plasma and exosomes from tumor-draining mesenteric vein	RT-qPCR	All members of the miR-200 family	Upregulated	Colon cancer	[207]
Serum	RT-qPCR	miR-141, -200a, -200b, and -200c	Upregulated	Cholangiocarcinoma	[222]
Serum exosomes	RT-qPCR	miR-200b, and -200c	Upregulated	Pancreatic ductal adenocarcinoma	[208]
Serum	TaqMan low density array	miR-200a, -200b, and -200c	Upregulated	Breast cancer	[204,205]
